# Myeloperoxidase Deficiency Alters the Process of the Regulated Cell Death of Polymorphonuclear Neutrophils

**DOI:** 10.3389/fimmu.2022.707085

**Published:** 2022-02-08

**Authors:** Silvie Kremserová, Anna Kocurková, Michaela Chorvátová, Anna Klinke, Lukáš Kubala

**Affiliations:** ^1^ Institute of Biophysics, Academy of Sciences of the Czech Republic, Brno, Czechia; ^2^ Department of Experimental Biology, Faculty of Science, Masaryk University, Brno, Czechia; ^3^ International Clinical Research Center, St. Anne’s University Hospital Brno, Brno, Czechia; ^4^ Clinic of General and Interventional Cardiology/Angiology, Agnes Wittenborg Institute of Translational Cardiovascular Research, Herz- und Diabeteszentrum NRW, Ruhr-Universität Bochum, Bad Oeynhausen, Germany

**Keywords:** myeloperoxidase, inflammation, neutrophils, apoptosis, cell death, annexin V

## Abstract

Polymorphonuclear neutrophils (PMNs) play a key role in host defense. However, their massive accumulation at the site of inflammation can delay regenerative healing processes and can initiate pathological inflammatory processes. Thus, the efficient clearance of PMNs mediated by the induction of regulated cell death is a key process preventing the development of these pathological conditions. Myeloperoxidase (MPO), a highly abundant enzyme in PMN granules, primarily connected with PMN defense machinery, is suggested to play a role in PMN-regulated cell death. However, the contribution of MPO to the mechanisms of PMN cell death remains incompletely characterized. Herein, the process of the cell death of mouse PMNs induced by three different stimuli – phorbol 12-myristate 13-acetate (PMA), opsonized streptococcus (OST), and N-formyl-met-leu-phe (fMLP) – was investigated. MPO-deficient PMNs revealed a significantly decreased rate of cell death characterized by phosphatidylserine surface exposure and cell membrane permeabilization. An inhibitor of MPO activity, 4-aminobenzoic acid hydrazide, did not exhibit a significant effect on PMA-induced cell death compared to MPO deficiency. Interestingly, only the limited activation of markers related to apoptotic cell death was observed (e.g. caspase 8 activation, Bax expression) and they mostly did not correspond to phosphatidylserine surface exposure. Furthermore, a marker characterizing autophagy, cleavage of LC3 protein, as well as histone H3 citrullination and its surface expression was observed. Collectively, the data show the ability of MPO to modulate the life span of PMNs primarily through the potentiation of cell membrane permeabilization and phosphatidylserine surface exposure.

## Introduction

The inflammatory process is primarily protective and vital to health. However, inflammation unrestrained in amplitude or duration leads to the development of pathological conditions and diseases. Although acute inflammation is generally self-limited, alternate fates include conversion to overwhelming systemic inflammation or chronic inflammation (1). One of the crucial steps in the final stage of the inflammation course is the clearance of polymorphonuclear neutrophils (PMNs) that massively infiltrate the site of inflammation (2).

PMN removal principally starts with the induction of regulated cell death in PMNs, followed by the uptake of dying cells by macrophages. The predominant regulated cell death of PMNs in this process is considered apoptosis (3, 4). The apoptosis of PMNs shares many morphological features with apoptosis in other cell types characterized by the exposure of phosphatidylserine (PS) on the outer leaflet of the cell membrane, the activation of caspases, DNA fragmentation, cytoplasmic condensation, membrane blebbing, and the formation of apoptotic bodies (5). PS externalization is considered a general phenomenon in cells undergoing apoptosis, although a few instances of apoptosis in the absence of PS exposure have also been described. Furthermore, during apoptosis, the activation of caspases is recognized to be crucial for the initiation, propagation, and execution of apoptosis (6). In general, the intrinsic pathway of apoptosis is regulated by Bcl-2 family proteins. PMNs constitutively express the proapoptotic proteins Bax, Bak, Bad, Bid, and Bik, which play an important role in PMN apoptosis. Bid is cleaved and activated by caspase-8, and the truncated Bid can then promote apoptosis by engaging its prosurvival Bcl-2-like relatives or by binding to the proteins Bak and Bax. The activation of Bax and Bak proteins leads to the induction of mitochondrial outer membrane permeabilization, caspase activation, and apoptosis (7).

Besides apoptosis, other forms of PMN cell death have also been described, including autophagy, NETosis, necrosis, and necroptosis [reviewed in (6, 8, 9)]. Autophagy is characterized by the modification of the microtubule-associated proteins 1A/1B light chain 3B (LC3) and p62, by massive cytoplasmic vacuolization, and by the absence of chromatin condensation (10, 11). In the case of LC3, the phosphatidylethanolamine-conjugated form of LC3, named LC3-II, is closely correlated with the number of autophagosomes and serves as an indicator of autophagosome formation. Autophagy has a complex relationship with various modes of cell death, including apoptosis and NETosis, and has also been implicated in caspase-independent cell death necrosis and necroptosis (12). Autophagy is associated with a response to cellular stressors (11) including the induction of reactive oxygen species (ROS) production or toll-like receptor activation and phagocytosis (13), suggesting both the phagocytosis-dependent and independent induction of autophagy in PMNs (14). Furthermore, the process termed NETosis, related to the formation of neutrophil extracellular traps (NETs) by PMNs, was described (15). The primary function of NETs seems to be the binding and killing of bacteria and fungi. However, NETosis is suggested to be associated with autophagy-related processes that lead to the release of nuclear DNA and mostly to PMN cell death. Interestingly, emerging evidence reveals that the post-translational modifications of histones have a critical role in regulating the form of PMN death when histone citrullination is involved in NET formation and NETosis, both dependent on, and independent of ROS production (16). Finally, PMNs can undergo necrosis morphologically characterized by a gain in cell volume, the swelling of organelles, plasma membrane rupture, and the subsequent loss of intracellular contents (6). Necrosis can proceed either as a primary event (primary necrosis) or can occur following apoptosis, when the macrophage-mediated phagocytosis of apoptotic corps is delayed (secondary necrosis). Conventionally, necrosis is considered a non-specific mode of cell death, but certain types of necrosis are specifically regulated and involve the modulation of the activity of proteins such as receptor-interacting serine/threonine-protein kinase 1 (RIP1), which is connected with RIP1 phosphorylation (17).

The production of ROS catalyzed primarily by phagocyte NADPH oxidase upon PMN activation by various stimuli is important for most of types of PMN cell death (7, 18–22). Indeed, PMNs from patients with chronic granulomatous disease, in which ROS generation is greatly impaired, exhibit delayed apoptosis, granuloma development, and the absence of PS externalization (21, 23–25). Various mechanisms were previously suggested for the ROS contribution to PMN-regulated cell death that include the destabilization of azurophilic granule membranes, the release of oxidants from destabilized granules, and the activation of proteases such as cathepsin D leading to caspase-8 activation (26, 27).

Interestingly, a molecule newly suggested to affect the cell death of PMNs is myeloperoxidase (MPO), which is responsible for the formation of highly reactive species after PMN stimulation. MPO is an abundant hemoprotein of PMNs, which is typically perceived primarily to mediate host defense reactions [reviewed in (19, 28, 29)]. However, there is increasing evidence of a novel regulatory role of MPO not directly related to host defense. MPO can significantly modulate redox-sensitive cellular signaling pathways controlling inflammatory processes through the catabolism of nitric oxide, the induction of a wide range of posttranslational modifications of proteins, and modulation of the metabolism of arachidonic and linoleic acid-derived mediators (19, 29, 30).

However, the importance of MPO in various inflammatory conditions is unclear due to its high dependence on the type of pathological inflammatory process. Consistent with the microbicidal effects of MPO-catalyzed reactive species, MPO deficient mice were more likely to be infected or die from infection employing various models, suggesting that the MPO-dependent oxidative system is important for host defense against some fungi and bacteria (19, 29, 31, 32). In contrast, in cases of inflammatory response to noninfectious stimuli, negative effects of MPO connected with damage to host tissue through the action of MPO-catalyzed oxidants are observed. These pro-inflammatory properties of MPO have been described in many chronic inflammatory diseases, including cardiovascular diseases, rheumatoid arthritis, and kidney diseases [see reviews (19, 29–31)]. Thus, on the basis of the assumption of the detrimental effect of MPO during chronic inflammation, it can be expected that inflammation should actually be reduced in MPO-deficient mice. However, experimental data from studies evaluating the pathological process of sterile and chronic inflammation in MPO-deficient mice revealed that MPO deficiency is connected with a dysregulated inflammatory response manifesting itself in paradoxically worse outcomes in MPO-deficient mice. Significant adverse effects of MPO deficiency were described in various models, including inflammatory pathological processes [see reviews (19, 29–31)]. These observations support the importance of a regulatory feedback role on the part of MPO in pathologies characterized by a complex inflammatory response in the absence of an infectious agent. However, these mechanisms are currently unclear. Previously, we demonstrated the importance of MPO as a mediator limiting the accumulation of PMNs in inflamed lungs, employing a model of endotoxin-induced lung inflammation characterized by an intensive increase in lung lavage cell numbers in both wild-type (WT) and MPO deficient mice that is almost exclusively due to PMNs (33). One of the proposed mechanisms was the altered cell death of MPO-deficient PMNs, which corresponded with suggestions by other authors suggesting the importance of MPO in different models of regulated cell death (20, 22, 34–37). However, the exact nature of the MPO-mediated activation of intracellular pathways characterizing the type of regulated cell death remains unclear.

On the basis of our previous study suggesting the delayed cell death of PMNs from lungs of MPO-deficient mice (33), we analyzed the mechanisms responsible for this phenomenon. We sought to examine differences in the main characteristics of regulated cell death between non-adherent PMNs isolated from the lungs of WT and MPO-deficient mice with sterile acute airway inflammation induced by endotoxin. The results suggest a new regulatory role on the part of MPO in the process of PS externalization and membrane permeabilization related to PMN cell death, which reveals the characteristics of different types of regulated cell death including apoptosis, autophagy, and NETosis.

## Materials and Methods

### Animal Exposure to LPS

A model of acute airway inflammation was elicited by the intranasal administration of lipopolysaccharide (LPS) (Escherichia coli serotype 055:B5, Sigma-Aldrich, St. Louis, MO, USA) inducing an inflammatory response with the extravasation of primarily PMNs in the airways (33, 38). Specifically, male C57BL/6J wild-type (WT) and MPO-deficient MPO_tm1lus (MPO^-/-^) mice (The Jackson Laboratory, USA), both aged 12-16 weeks and weighing 25-30 g, were subjected to brief anesthesia with ketamine-xylazine, after which 50 µl of LPS solution in phosphate-buffered saline (PBS) was instilled directly into the nostrils to achieve a dose of 0.3 mg/kg LPS, as described previously (33, 38). Previous studies demonstrated that a significant fraction of intranasally administered LPS reaches the lungs, and that such instillation evokes an acute transient inflammatory response (33, 38). All animal experiments were conducted in accordance with the EU Guide for the Care and Use of Laboratory Animals, and the experimental protocol was approved by the institutional Animal Care and Use Committee (The Czech Academy of Sciences of the Czech Republic, protocol n. 42/2015 from 12^th^ June 2015).

### Collection of PMNs From Bronchoalveolar Lavage Fluid and PMN Treatments

Mice were deeply anesthetized by the intraperitoneal administration of ketamine-xylazine 48 hours after LPS instillation. The tracheae were cannulated, and the lungs were lavaged with 3 consecutive washes with 1 ml of PBS, which were pooled to a total recovered volume of bronchoalveolar lavage fluid (BALF) of 2.5 - 2.7 ml. Total cell counts in the collected lavage samples were determined by means of a Casy Cell Counter and Analyzer (Roche Diagnostic, Indianapolis, IN, USA) after lysis of contaminating red blood cells by Ekoglobin (Hemax s.r.o., Czech Republic). As previously described, almost 95% of all cells in BALF were PMNs (33, 38).

PMNs were diluted to a specific concentration (3x10^6^ – 1x10^7/^ml) in HEPES buffered RPMI 1640 (ThermoFisher Scientific, Waltham, MA, USA) supplemented with 0.1% gelatin (G9391, Sigma-Aldrich), and directly processed for analysis (untreated control without incubation), incubated without stimulation (control groups, ctrl), or stimulated by phorbol-12-myristate-13-acetate (PMA, 80 nM, in the case of chemiluminescence (CL) 800 nM) (Sigma-Aldrich), opsonized Streptococcus mutans (OST) (3x10^7^ - 1x10^8^ bacteria/ml), and N-Formylmethionyl-leucyl-phenylalanine (fMLP) (Sigma-Aldrich) (1.14 μM) for 90 or 180 min. In specific cases as a control, sample cells were incubated in the medium without activation for 24 h. As a positive control for specific cell death types, PMNs were incubated with tumor necrosis factor α (TNF-α) (10 ng/ml) (Peprotech, USA) or irradiated by UV light for 20 min. In selected cases, PMNs were treated with hypochlorus acid (HOCl) (Sigma-Aldrich) in the concentration range 0.15 mM - 10 mM. In selected cases, PMNs were pretreated by externally-added purified human MPO (Planta Natural Products, Austria) (10 μg/ml) or by the MPO inhibitor 4-Aminobenzoic acid hydrazide (4-ABAH) (Sigma-Aldrich) at concentrations of 50, 100, 250 and 500 µM 10 min before the addition of PMA.

*Streptococcus mutans* (Czech Collection of Microorganisms, Brno, Czech Republic) was cultured in liquid medium (1 mg/ml brain heart infusion; 1 mg/ml bacteriological peptone; 0.5 mg/ml NaCl) at 37°C with agitation overnight. Bacteria achieving logarithmic growth were washed with saline solution (0.9% NaCl) (3 255 g, 10 min, RT) and suspended in Hanks’ Balanced Salt solution (HBSS) to reach an absorbance of 1.1 at 400 nm, which correlated with a cell density of 3x10^9^ cells/ml. The suspension was mixed with pooled mouse serum (2 parts suspension and 1 part mouse serum) and opsonized in a water bath at 37°C for 30 min with continuous shaking. Subsequently, the cells were washed twice in saline (3 255 g, 10 min, RT), resuspended in HBSS to reach a concentration of 3x10^9^ cells/ml, and frozen in aliquots at -20°C for further use.

### Detection of Protein Expression by Western Blot Technique

After treatment, the cells were lysed using sodium dodecyl sulfate (SDS)-lysing buffer. The same amount of protein (25 µg) from each lysate was subjected to 10% SDS-polyacrylamide gel electrophoresis and blotted on a poly(vinylidene fluoride) membrane (Bio-Rad), as previously described (39). Membranes were blocked by 5% skimmed milk (for Bid, Bax, GAPDH), 3% bovine serum albumin (for LC3), 5% bovine serum albumin (for P62, RIP1), and 1% fish skin gelatine (for histone), and then incubated with primary antibodies against Bax, p62, RIP1 (Cell Signaling Technology, Danvers, MA, USA), Bid (R&D System, Minneapolis, MN, USA), citrullinated histone H3 and histone H3 (Abcam, UK), LC3 (L8918, Sigma-Aldrich), and GAPDH (14C10, Cell Signaling Technology) overnight. Horseradish peroxidase conjugated goat anti-rabbit antibody (1:5 000; Santa Cruz Biotechnology, USA) was used as a secondary antibody. The blots were visualized using SuperSignal West Pico Chemiluminescent Substrate (Pierce, USA) and exposed to CP-B X-ray films (Agfa, Czech Republic) or scanned by an Amersham Imager 680 (General Electric Company, USA). The relative levels of proteins were quantified by scanning densitometry using ImageJ™ software, and the individual band density values were expressed in arbitrary units (optical density, OD). The loading controls are provided to show an equality of the total protein loading. The OD of the investigated bands was not normalized to the OD of the loading controls or to the OD of the total protein.

### Caspase Activity Assay

Caspase activity was analyzed as described previously (33). Isolated cells were washed twice with PBS, lysed (50 mM HEPES; 5 mM CHAPS; 5 mM dithiothreitol) (all Sigma-Aldrich) on ice for 20 min, and centrifuged (15 000 g for 15 min) at 4°C. Lavage fluid was diluted 1:1 with 2x concentrated lysing buffer. The proteins present in supernatants were quantified using Coomassie Protein Assay (Bio-Rad, California, USA) and diluted to the same concentration. 5 µg of protein samples were incubated in an assay buffer (20 mM HEPES; 2.5 mM CHAPS, 5 mM dithiothreitol, 2 mM EDTA) containing 50 µM of caspase 3 (Ac-DEVD-AMC) or caspase 8 substrate (Sigma-Aldrich) at 37°C for 4 h. The level of fluorescence was determined using a microplate reader (Infinite 200, Tecan, Switzerland; excitation – 360 nm for caspase 3 or 390 nm for caspase 8, emission - 460 nm for caspase 3 or 535 nm for caspase 8).

### Phosphatidylserine PS Externalization (Annexin V/Propidium Iodide Assay) and Determination of Surface Citrullinated Histone H3

The presence of cells with permeable membranes (dead cells) and cells with a surface expression of PS was evaluated by flow cytometry using propidium iodide (PI) and Annexin V (ANX) staining, respectively (33). After treatment, cells were washed with PBS, resuspended in 100 μl of Annexin V binding buffer (10 mM HEPES/NaOH, pH 7.4, 140 mM NaCl, 2.5 mM CaCl_2_), and incubated for 20 min with 1 μl Annexin V-FITC (Apronex Ltd, Czech Republic). PI (1 μg/ml) was added 1 min before analysis. Flow-cytometric analysis of the stained cells was performed using a BD FACSVerse™ flow cytometer (BD Biosciences, San Jose, CA USA). The cell population for the analysis was gated using forward- versus side-scatter parameters to exclude any debris. Per sample, 10 000 cells were collected. Three different populations can be identified using this assay: intact viable cells that are negative for both PI and Annexin V-FITC, cells positive for Annexin V-FITC but negative for PI, and permeable cells positive for both Annexin V-FITC and PI.

To determine the presence of citrullinated histone H3 on the surface of cells, PMNs were diluted to a concentration of 3x10^6^/ml and divided into flow-cytometric tubes (100 µl of suspension/tube). After incubation with activators for 180 min, cells were stained with primary antibody (1:300) histone H3 (Abcam, UK) for 10 min. Afterwards, fluorescently-labeled secondary antibody Goat anti-rabbit IgG, DyLight 649 (Thermo Fisher Scientific, USA) was added (1:3000) and samples were incubated for 20 min at RT, protected from light. Fluorescence was measured using a BD FACSVerseTM flow cytometer (BD Biosciences, San Jose, CA USA). The cell population for the analysis was gated using forward- versus side-scatter parameters to exclude any debris. Per sample, 10 000 cells were collected. Data analysis was performed using Flowing Software (www.flowingsoftware.com).

### DNA Fragmentation

3x10^6^ cells isolated from BALF were washed with PBS, and DNA was isolated using the Invisorb Apoptosis Detection Kit II (Invitek; Invitek GmbH, Germany). Gel electrophoresis was performed in 1.5% agarose (Sigma-Aldrich) using a 1 kbp DNA ladder as a marker (ThermoFisher Scientific). DNA was stained with Ethidium bromide (Sigma-Aldrich) and scanned on a UV-transilluminator using Scion Image software (Scion Corporation, USA).

### Analysis of PMN Oxidative Burst by Luminol Dependent Chemiluminescence

Reactive oxygen species formation during the oxidative burst of PMNs was determined by luminol-enhanced CL employing a peroxidase-sensitive luminol probe, as described previously (38). For this experiment, the concentration of PMNs was adjusted to 1.25 x 10^6^ cells/ml. Luminol (10 μl) (a 1 mM stock solution of 10mM luminol was prepared in 0.2 M borate buffer), 80 μl of cell suspension, and 10 μl of activator were pipetted into a 96-well microtiter plate. Spontaneous CL in samples containing all other substances, but none of the activators, were also determined. For measurements of CL in combination with a 4-ABAH inhibitor, the inhibitor was first pipetted (50, 100, 250, and 500 µM) before the cells were stimulated. CL measurements were performed on an LM-01T luminometer (Immunotech, Czech Republic) for 120 min at 37°C. The values of the integral measurements of the CL signal (the area under the curve), which represented the total production of ROS by PMNs, were determined for further statistical analysis.

### Statistical Analysis

For multiple comparisons, one-way ANOVA followed by the Dunnett *post hoc* test, or unpaired t-tests with Bonferroni correction of the p-value for multiple comparisons were used as appropriate. A *p* value equal to or lower than 0.05 was considered statistically significant. Data are presented as “box and whiskers” plots showing median, 25, and 75 percentiles (the box), and 10 and 90 percentiles (the whiskers); values below and above the whiskers are drawn as individual dots; or all individual values are drawn in the cases of OD analysis. All statistical analyses were carried out using GraphPad Prism 6 Software (GraphPad Software, San Diego, CA).

## Results

### MPO Deficiency Delays the Surface Expression of PS on PMNs

**Figure 1:**
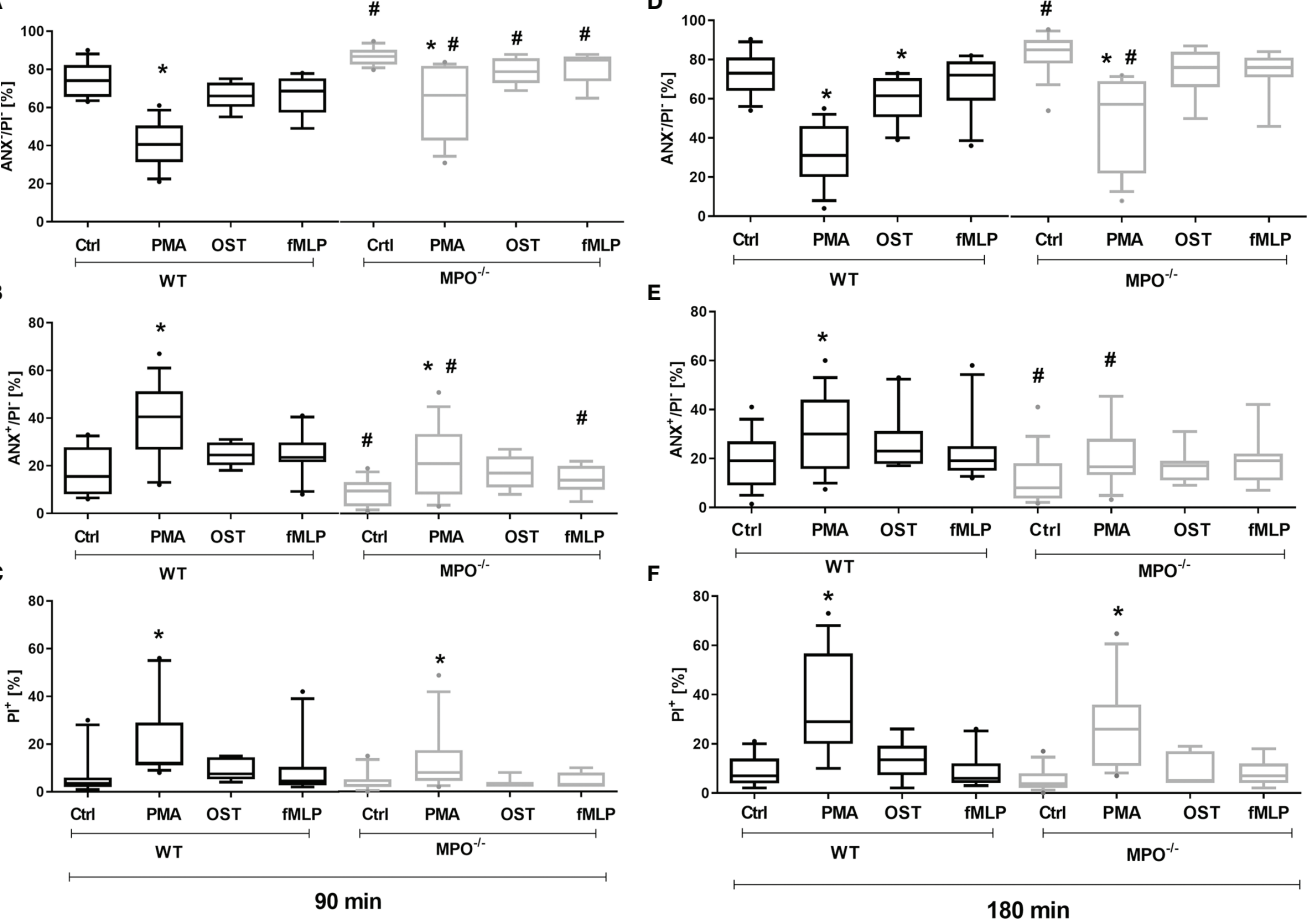
Increased Annexin V^+^ and cell death (PI^+^) of PMNs isolated from inflamed lungs of WT and MPO^-/-^ mice after the induction of oxidative burst by PMA, OST, and fMLP for 90 min **(A–C)** and 180 min **(D–F)**. PMNs (3x 10^6^ cells/ml) isolated from BALF of WT and MPO^-/-^ mice were stimulated with PMA (80 nM), OST (1:10 MOI) and fMLP (1.14 μM) for 90 and 180 min. Next, cells were evaluated by flow cytometry and numbers of viable (ANX^-^/PI^-^), Annexin V^+^ (ANX^+^/PI^-^), and dead cells (PI^+^, both ANX^+^/PI^+^ and ANX^-^/PI^+^) were determined. Data are presented as median, 25, and 75 percentiles (the box) and 10 and 90 percentiles (the whiskers), and values below and above the whiskers are drawn as individual dots, n = 7-14. *Shows statistically significant difference (p < 0.05) compared with untreated control (Ctrl). # shows statistically significant difference (p < 0.05) between MPO^-/-^ (MPO-KO) and wild-type (WT).

To evaluate the effect of MPO-derived oxidants in the course of PMN cell death, PMNs isolated from lung lavage of WT and MPO^-/-^ mice were stimulated by selected activators of oxidative burst, specifically PMA, OST, and fMLP. The stimulation of WT PMNs with PMA significantly increased the number of Annexin V^+^ PMNs (**Figures 1B, E**), with PS on the surface, and PI^+^ PMNs (**Figures 1C, F**), with permeable membranes, these increases accompanied by a decrease in the number of viable cells, negative for either Annexin V or PI both after 90 min and 180 min (**Figures 1A, D**). Characteristic scattered plots from flow-cytometric analysis are depicted in **Supplementary Figure 1**. The stimulation of WT PMNs with OST and fMLP increased the number of Annexin V^+^ cells only marginally and without statistical significance compared to unstimulated control (**Figures 1A–F**). In contrast, MPO^-/-^ PMNs revealed a higher number of viable cells compared to WT PMNs after 90 min of incubation, independently of stimulation (**Figures 1A**). Correspondingly, the numbers of Annexin V^+^ PMNs without stimulation and stimulated by PMA and fMLP were lower compared to WT PMNs at this time point (**Figure 1B**). After 180 min of incubation, a significantly higher number of viable cells and a significantly lower number of Annexin V^+^ MPO^-/-^ PMNs compared to WT PMNs were observed in unstimulated control as well as in PMA-stimulated cells (**Figures 1D, E**).

**Figure 2:**
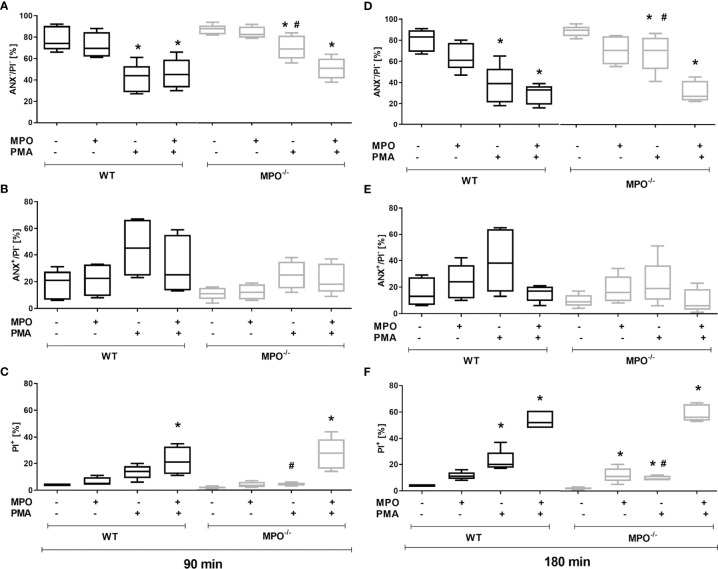
Effect of extracellularly-added MPO on Annexin V^+^ and cell death (PI^+^) of PMNs isolated from inflamed lungs of WT and MPO^-/-^ mice after the induction of oxidative burst by PMA for 90 min **(A–C)** and 180 min **(D–F)**. PMNs (3x 10^6^ cells/ml) isolated from BALF of WT and MPO^-/-^ mice were pre-treated with/without MPO (20 µg/ml), then stimulated with PMA (80 nM) for 90 and 180 min. Next, cells were evaluated by flow cytometry and numbers of viable (ANX^-^/PI^-^), Annexin V^+^ (ANX^+^/PI^-^), and dead cells (PI^+^, both ANX^+^/PI^+^ and ANX^-^/PI^+^) were determined. Data are presented as median, 25, and 75 percentiles (the box) and 10 and 90 percentiles (the whiskers), n = 4–6. *Shows statistically significant difference (p < 0.05) compared with untreated control (Ctrl). # shows statistically significant difference (p < 0.05) between MPO^-/-^ (MPO-KO) and wild-type (WT).

To confirm the direct role of MPO in the potentiation of PS surface expression and cell membrane permeability in spontaneously- or PMA-stimulated dying PMNs, MPO purified from human leukocytes was added externally to isolated PMNs before incubation. In accord with our hypothesis, the addition of MPO to PMA-stimulated PMNs from MPO^-/-^ mice significantly promoted a decrease in cell viability and an increase in PI^+^ cell number after both 90 and 180 min (**Figures 2A, C, D, F**). As a consequence, the number of viable and PI^+^ cells in PMA-stimulated WT and MPO^-/-^ PMNs no longer differed significantly. Moreover, the addition of MPO even further promoted the number of PI^+^ WT PMNs after PMA stimulation for 180 min (**Figure 2F**). Interestingly, the addition of MPO also promoted an increase in PI^+^ MPO^-/-^ PMNs incubated without stimulation for 180 min (**Figure 2F**). In contrast, PS externalization was not enhanced by the addition of external MPO (**Figures 2B, E**). Next, the effects of the direct addition of HOCl on PMN viability and PS surface exposure were tested in the concentration range 0.15 mM - 10 mM for a period of up to 180 min. Interestingly, externally-added HOCl did not induce a significant increase in Annexin V^+^ PMNs in either WT or MPO^-/-^. Specifically, HOCl at concentrations of 0.15 or 0.3 mM did not reveal any effects on the numbers of viable Annexin V^+^ or PI^+^ PMNs, whereas treatment by 1.5 – 10 mM HOCl induced a massive increase in PI^+^ PMNs without the formation of an Annexin V^+^ population (data not shown). Results obtained with 1 mM HOCl revealed high variability and were inconclusive: in some cases, 1 mM HOCl did not reveal any effects on the numbers of viable Annexin V^+^ or PI^+^ PMNs, similarly to treatment without HOCl and similarly to concentrations of 0.15 - 0.3 mM, while in some cases a substantial increase in PI^+^ PMNs without the formation of an Annexin V^+^ population was observed (data not shown).

To further evaluate the influence of MPO enzymatic activity on the observed phenomenon, the effect of MPO inhibitor 4-ABAH (500 µM) was tested. Interestingly, this MPO inhibitor did not have any significant effect on the numbers of Anexin V^+^, PI^+^, or viable WT PMNs, either PMA-stimulated or incubated without activation for 90 or 180 min (**Supplementary Figure 2**). Similar results were also obtained for the other two activators, OST and fMLP (data not shown). To confirm the expected inhibitory effect of 4-ABAH on peroxidase-dependent ROS formation, we performed the peroxidase-sensitive determination of ROS by luminol-enhanced CL. The effects of 4-ABAH were tested in the concentration range of 50 to 500 µM. Interestingly, 4-ABAH decreased the CL signal from both spontaneous and PMA-stimulated PMNs; however, none of these concentrations decreased the CL signal to the background level, i.e. prevented peroxidase activity completely (**Supplementary Figure 3A**). To confirm the relevance of the CL method for the determination of MPO activity, the CL signal obtained after stimulation by all tested activators was compared between WT and MPO^-/-^ PMNs. This confirmed the expected orders-of-magnitude-higher CL signal from WT PMNs compared to MPO^-/-^ PMNs (**Supplementary Figures 3B, C**). The signal obtained from unstimulated control PMNs and OST- and fMLP-stimulated MPO^-/-^ PMNs was at the background level. However, MPO^-/-^ PMNs stimulated with PMA revealed detectable CL levels, suggesting the potential of MPO^-/-^ PMNs to oxidize luminol despite MPO deficiency. Consequently, 4-ABAH significantly decreased only CL produced by WT PMNs, either spontaneous CL or that stimulated by PMA, OST, and fMLP, and did not have any significant effect on CL produced by MPO^-/-^ PMNs (**Supplementary Figures 3B, C**).

### Modulation of Classical Apoptotic Markers Including Caspase 3 and 8 Activation, Bid and Bax Cleavage, DNA Fragmentation, as Well as the Expression and Phosphorylation of RIP1

**Figure 3:**
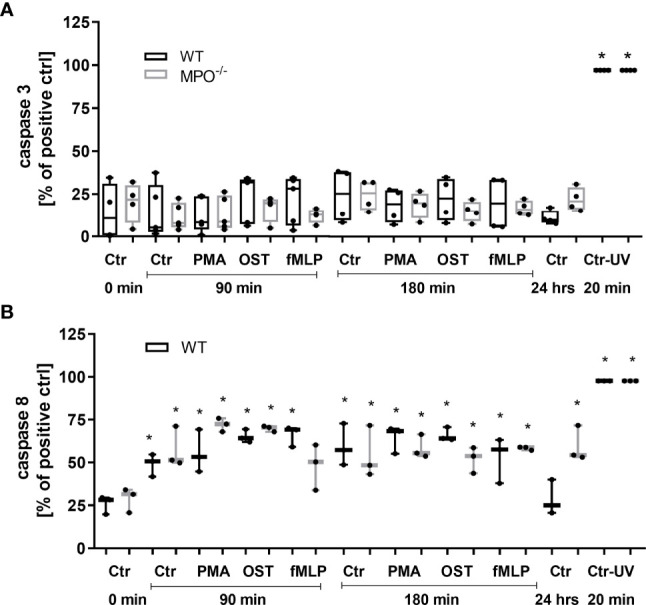
Activity of caspase 3 **(A)** and 8 **(B)** in PMNs isolated from inflamed lungs of WT and MPO^-/-^ mice after the induction of oxidative burst by PMA, OST, and fMLP for 90 min and 180 min. PMNs (1x 10^7^ cells/ml) isolated from BALF of WT and MPO^-/-^ mice were stimulated with PMA (80 nM), OST (1:10 MOI) and fMLP (1.14 μM) for 90 and 180 min. PMNs incubated for 24 h without stimulation and PMNs irradiated by UV for 20 min were also used as a positive control. Next, cells were lysed and caspase 3 or caspase 8 activity was determined by enzymatic assay. Data are presented as median, 25, and 75 percentiles (the box) and 10 and 90 percentiles (the whiskers), and individual values are drawn as individual dots, n = 3 - 4. *Shows statistically significant difference (p < 0.05) compared with untreated control (Ctrl).

**Figure 4:**
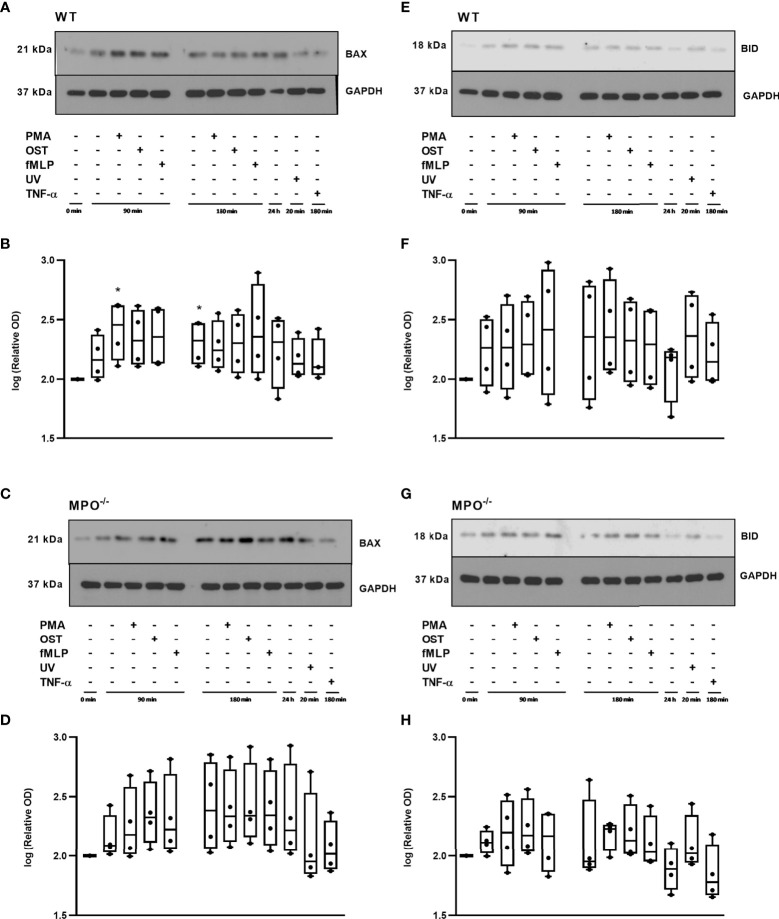
Apoptotic proteins Bax **(A-D)** and Bid **(E-H)** in PMNs isolated from inflamed lungs of WT **(A, B, E, F)** and MPO^-/-^ **(C, D, G, H)** mice after induction of oxidative burst by PMA, OST, and fMLP for 90 min and 180 min. PMNs (1x 10^7^ cells/ml) isolated from BALF of WT and MPO^-/-^ mice were stimulated with PMA (80 nM), OST (1:10 MOI) and fMLP (1.14 μM) for 90 and 180 min. PMNs incubated for 24 h without stimulation, PMNs irradiated by UV for 20 min, and PMNs treated by TNF-α (10 ng/ml) for 180 min were tested as positive controls. Proteins Bax, Bid and GAPDH (as a marker of equal loading) were determined by Western blot. Analysis of OD was performed using ImageJ, and values are depicted as percentage of untreated control transformed by Log2 to normalize the data distributions **(B, D, F, H)**. Data are presented as median, 25, and 75 percentiles (the box) and 10 and 90 percentiles (the whiskers), and individual values are drawn as individual dots, n = 4. *Shows statistically significant difference (p < 0.05) compared with untreated control (Ctrl). Figures **(A, C, E, G)** represent typical examples.

To further analyze the mechanisms responsible for the MPO-mediated potentiation of PMN cell death, the analysis of caspase 3 and 8 activation and Bid and Bax expression was performed, as these are generally accepted markers of apoptotic cell death. In contrast to increased Annexin V positivity, no significant increase in caspase 3 activity was found in any type of stimulated WT or MPO^-/-^ PMNs compared to untreated control PMNs without any incubation, reaching approximately 20 - 25% of the activity of the positive control, the UV-treated PMNs (**Figure 3A**). Caspase 3 cleavage by western blot was not detectable in these samples (data not shown). Interestingly, in contrast, the activity of caspase 8 was significantly increased in both unstimulated as well as stimulated WT and MPO^-/-^ PMNs incubated for 90 or 180 min compared to untreated control PMNs without any incubation, and, overall, reached approximately 50-75% of the activity of the positive control, the UV treated PMNs (**Figure 3B**). In the case of Bax protein, a significant increase in total expression was observed in the case of WT PMNs stimulated by PMA for 90 min and unstimulated WT PMNs incubated for 180 min (**Figures 4A, B**). However, there was also an indication of an increase in total Bax expression in other stimulated WT and MPO^-/-^ PMNs, albeit without statistical significance (**Figures 4A–D**). However, there was no indication of Bax fragmentation in any sample. Concerning the other typical apoptotic protein Bid, there were no significant changes in total expression or signs of fragmentation in either stimulated or any control WT or MPO^-/-^ PMNs, e.g. PMNs incubated for 24 h without stimulation, PMNs irradiated by UV for 20 min, or PMNs treated by TNF-α (10 ng/ml) for 180 min (**Figures 4E–H**). Similarly, another marker of the apoptotic process, DNA fragmentation, was not observed in PMA stimulated WT or MPO^-/-^ PMNs (data not shown).

**Figure 5:**
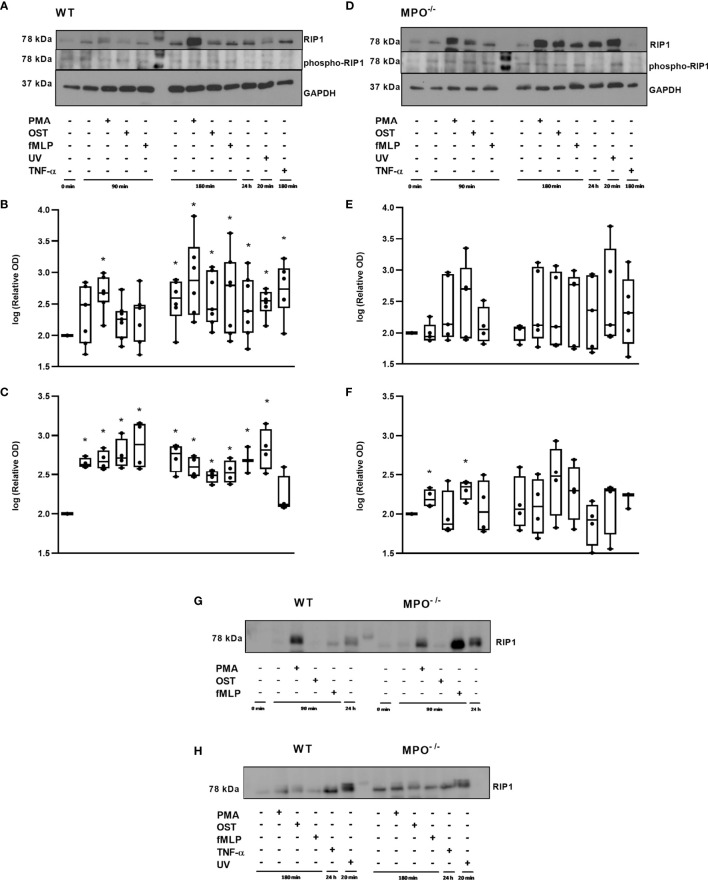
Expression of total and phosphorylated RIP1 in PMNs from WT **(A–C, G, H)** and MPO^-/-^ **(D–F, G, H)** mice after the induction of oxidative burst by PMA, OST, and fMLP for 90 min and 180 min. PMNs (1x 10^7^ cells/ml) isolated from BALF of WT and MPO^-/-^ mice were stimulated with PMA (80 nM), OST (1:10 MOI) and fMLP (1.14 μM) for 90 and 180 min. PMNs incubated for 24 h without stimulation, PMNs irradiated by UV for 20 min, and PMNs treated by TNF-α (10 ng/ml) for 180 min were tested as positive controls. Total and phosphorylated protein RIP1 and GAPDH (as a marker of equal loading) were determined by Western blot. Analysis of OD was performed using ImageJ, and values are depicted as percentage of untreated control transformed by Log2 to normalize the data distributions **(B, C, E, F)**. Data are presented as median, 25, and 75 percentiles (the box) and 10 and 90 percentiles (the whiskers), and individual values are drawn as individual dots, n = 3-5. *Shows statistically significant difference (p < 0.05) compared with untreated control (Ctrl). Figures **(A, D, G, H)** represent typical examples.

Further, RIP1 expression as well as phosphorylation related to the activation of this kinase were evaluated. Interestingly, total levels of RIP1 were significantly increased in WT PMNs stimulated by PMA for 90 min and also in WT PMNs from all types of treatments incubated for 180 min, as well as in the 24h control compared to control PMNs lysed shortly after isolation (**Figures 5A, B**). In the case of MPO^-/-^ PMNs, the trend in total RIP1 expression was similar, though less robust and without statistical significance compared to the untreated PMNs control (**Figures 5D, E**). The comparison of changes in total RIP1 level between WT and MPO^-/-^ PMNs confirmed a similar trend (**Figures 5G, H**). The analysis of phosphorylated RIP1 revealed a very weak signal that did not correspond to the total levels of RIP1 in particular PMNs isolated either from WT or MPO^-/-^ mice (**Figures 5A, D**). A densitometric analysis showed significantly increased levels of phosphorylated RIP1 in all WT PMN samples except the TNF-α treated one compared to untreated control (**Figures 5A, C**). In contrast, increased levels of phosphorylated RIP1 in MPO^-/-^ PMNs were mostly random and were significantly increased in the case of control PMNs incubated for 90 min and PMA stimulated for 90 min compared to untreated control (**Figures 5D, F**). However, the signal for phosphorylated RIP1 has to be treated with caution due to the limited intensity of the signal.

### Changes in the Expression of LC3 and p62, Histone H3 Citrullination, and Citrullinated Histone H3 Surface Expression

**Figure 6:**
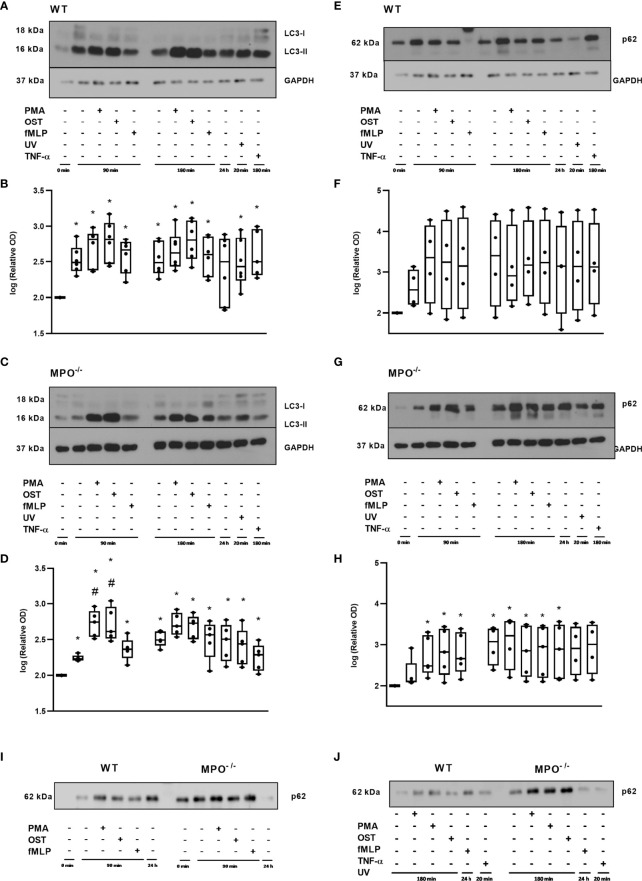
Autophagic proteins LC3 **(A–D)** and p62 **(E–J)** in PMNs from WT **(A, B, E, F, I, J)** and MPO^-/-^ **(C, D, G–J)** mice after the induction of oxidative burst by PMA, OST, and fMLP for 90 min and 180 min. PMNs (1x 10^7^ cells/ml) isolated from BALF of WT and MPO-/- mice were stimulated with PMA (80 nM), OST (1:10 MOI) and fMLP (1.14 μM) for 90 and 180 min. PMNs incubated for 24 h without stimulation, PMNs irradiated by UV for 20 min, and PMNs treated by TNF-α (10 ng/ml) for 180 min were tested as positive controls. Proteins LC3, p62 and GAPDH (as a marker of equal loading) were determined by Western blot. Analysis of OD was performed using ImageJ, and values are depicted as percentage of control PMNs (untreated and non-incubated) transformed by Log2 to normalize the data distributions **(B, D, F, H)**. Data are presented as median, 25, and 75 percentiles (the box) and 10 and 90 percentiles (the whiskers), and individual values are drawn as individual dots (n = 4-5). * shows statistically significant difference (p < 0.05) compared with untreated control (Ctrl). ^#^Shows statistically significant difference (p < 0.05) compared with unstimulated control incubated for the same time period.A comparions of p62 protein in PMNs from WT and MPO^-/-^ PMNs after the induction of oxidative burst by PMA, OST, and fMLP for 90 min **(I)** and 180 min **(J)**. Figures **(A, C, E, G, I, J)** represent typical examples.

Further, since, in general, the MPO-dependent increase in the rate of cell death of PMNs observed in our study did not reveal typical apoptotic cell death, an alternative type of cell death such as autophagy was investigated by employing LC3-II, as an indicator of autophagosome formation, and p62, as an indicator of p62-containing aggregates of ubiquitylated proteins. A significant increase in LC3-II compared to the untreated control of freshly-isolated PMNs without any incubation was observed in both WT and MPO^-/-^ PMNs in all groups including both unstimulated and PMA-, OST-, and fMLP-stimulated PMNs after 90 and 180 min, respectively (**Figures 6A–D**). Interestingly, the analysis of MPO^-/-^ PMNs revealed an even further significant increase in LC3-II compared to unstimulated control after 90 min of incubation (**Figures 6C, D**). In contrast, p62 levels were observed to be significantly increased compared to untreated control only in the case of MPO^-/-^ PMNs incubated for 90 min and 180 min with or without stimulation, as well as in PMNs cultured for 24 hours (**Figures 6G, H**). In the case of WT PMNs, there was only an indication of an increase in p62 expression compared to untreated control in all types of samples; however, this increase did not reach statistical significance due to the higher deviation among various independent replicates (**Figures 6E, F**). However, the comparison of changes in p62 levels between WT and MPO^-/-^ PMNs confirms a similar trend (**Figures 6I, J**).

**Figure 7:**
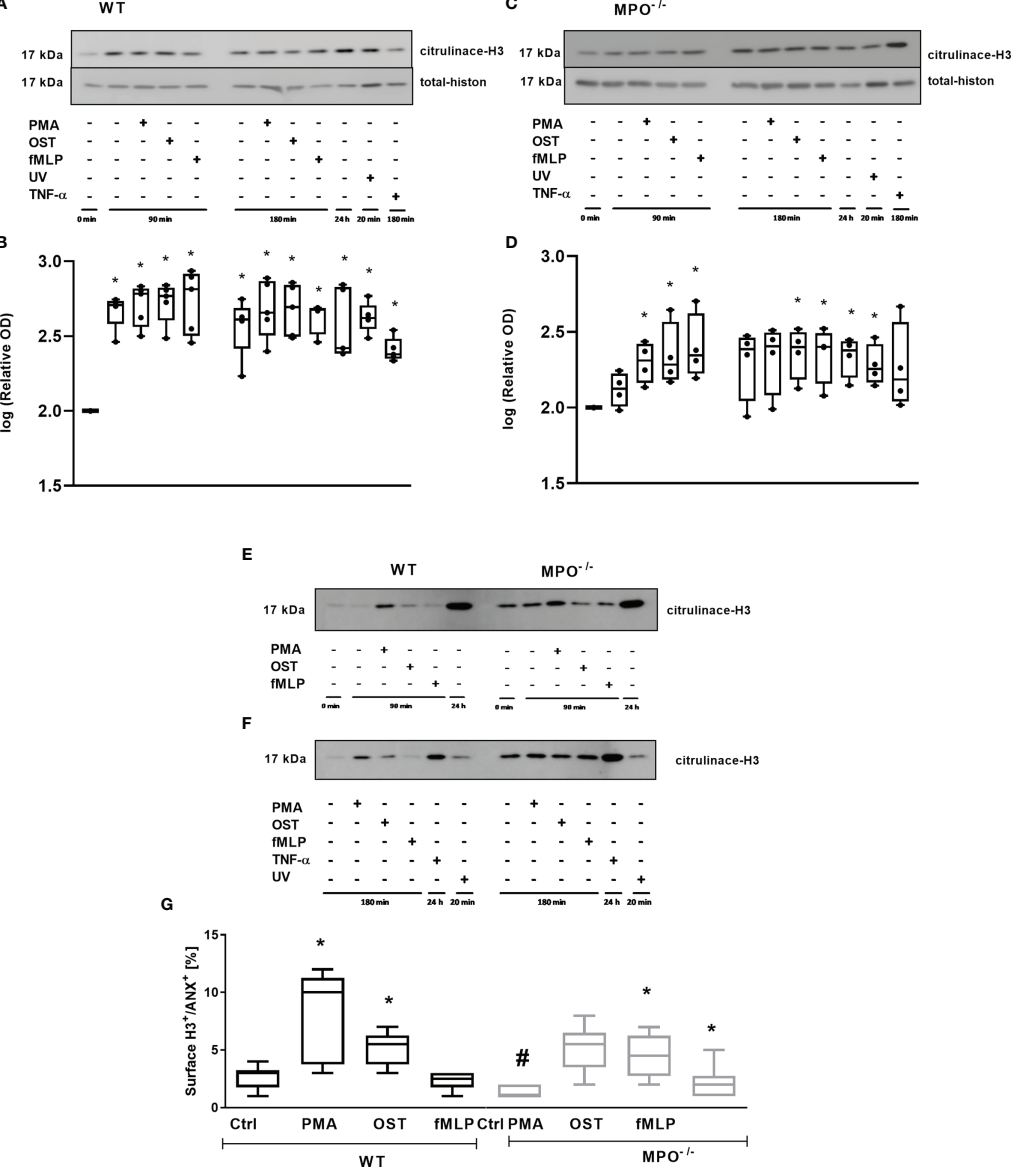
Determination of histone H3 citrullination in PMNs from WT **(A, E, F)** and MPO^-/-^ **(B, E, F)** and its surface expression on Annexin V^+^ PMNs **(G)**. **(A–F)** - PMNs (1x 10^7^ cells/ml) isolated from BALF of WT and MPO^-/-^ mice were stimulated with PMA (80 nM), OST (1:10 MOI) and fMLP (1.14 μM) for 90 and 180 min. PMNs incubated for 24 h without stimulation, PMNs irradiated by UV for 20 min, and PMNs treated by TNF-α (10 ng/ml) for 180 min were tested as positive controls. Total and citrullinated H3 histone was determined by Western blot. Data are presented as median, 25, and 75 percentiles (the box) and 10 and 90 percentiles (the whiskers), and individual values are drawn as individual dots (n = 3). * shows statistically significant difference (p < 0.05) compared with untreated control (Ctrl). A comparions of histone H3 cintrulination in PMNs from WT and MPO^-/-^ after stimulation with PMA, OST, and fMLP for 90 min **(E)** and 180 min **(F)**. **(G)** – PMNs were evaluated by flow cytometry and cells positive for both Annexin V^+^ and citrullinated H3^+^ were determined after 80 min. n = 3-4 mice per group. Data are presented as median, 25, and 75 percentiles (the box) and 5 and 95 percentiles (the whiskers), and values below and above the whiskers are drawn as individual dots. *Shows statistically significant difference (p < 0.05) compared with untreated control (Ctrl). # shows statistically significant difference (p < 0.05) between MPO^-/-^ (MPO-KO) and wild-type (WT).

In addition, we analyzed the importance of MPO for histone H3 citrullination in PMNs, since this posttranslational modification is generally related to PMN activation and also to Netosis. Extensive citrullination of H3 was observed in the case of WT PMNs in all types of samples without any differences between stimulated and unstimulated PMNs for either time points compared to the untreated control – specifically, freshly isolated PMNs without any incubation (**Figures 7A, B**). Interestingly, a similar trend was observed in MPO^-/-^ PMNs; however, samples with less extensive citrullination and with higher deviation, which were the control PMNs incubated for 90 min and 180 min, as well as PMA stimulated PMNs for 180 min, did not reach statistical significance compared to untreated control (**Figures 7C, D**). An example of the direct comparison of trends in H3 citrullination between WT and MPO^-/-^ PMNs is depicted in **Figures 7E, F** and confirms similar trends, despite the relative level of H3 citrullination appearing higher in MPO^-/-^ PMNs in these particular samples. Overall, the level of H3 citrullination was not dependent on the presence of MPO in stimulated PMNs. Finally, the surface presence of histone H3, which should be present on the surface due to the regulated release of genomic DNA during NETosis, was determined. Interestingly, in this case, we observed a significant increase in Histone 3 on the surface of WT PMN after PMA and OST activation for 180 min (**Figure 7G**). In the case of MPO^-/-^ PMNs, an increase in surface citrullinated H3 was also observed; however, due to higher deviation, this was significant only in the case of OST- and fMLP- stimulated PMNs (**Figure 7G**). Interestingly, the basal level in control non-stimulated PMNs was higher in WT PMNs compared to MPO^-/-^ PMNs.

## Discussion

This study presents the importance of MPO in altering the cell membrane integrity of PMNs activated *in vitro*, which was demonstrated by increased surface PS expression and cell membrane permeability. MPO-dependent alterations of PS in PMN membranes did not correlate with changes in the markers of classical apoptotic processes, selected markers of autophagy, or histone 3 citrullination. Interestingly, the role of MPO enzymatic activity based on 4-ABAH inhibition in the alteration of the cell membrane integrity of PMNs in this model was not confirmed and remains to be further explored.

The importance of MPO in the induction of cell death presenting with extensive PS exposure and cell membrane permeabilization was previously supported by other authors – however, with different conclusions. Milla et al. suggested that MPO deficiency is connected with suppressed PMN-regulated cell death (34). Similarly, Tsurubuchi at al. demonstrated delayed PS externalization in PMA-stimulated PMNs from MPO^-/-^ mice, in contrast to PMNs from WT mice (20). However, in their other study employing zymosan as a stimulator, they found no obvious difference in cell death between WT and MPO^-/-^ PMNs (35). Similarly, in contrast to NADPH-generated ROS, there was no requirement for MPO in the PMN externalization of PS in PMN-ingesting *Staphylococcus aureus* (22). In this study, the most significant effects were observed with PMA. This could be related to the higher potential of PMA to induce quick and robust oxidative burst compared to OST- and fMLP-stimulated oxidative burst.

In general, the type of PMN cell death highly depends on the activator and the conditions under which PMNs are stimulated. PS surface exposure is traditionally recognized as one of the earliest markers of apoptosis. However, changes in other markers including an increase in caspase 8 activity as well as some increase in total Bax protein levels and the indication of an increase in Bid protein, which were, however, in the majority of cases not statistically significant, did not correspond with the increases in surface PS and cell permeability in PMNs, particularly after PMA stimulation. Caspase 3 activity did not increase compared to unstimulated control and DNA fragmentation was not observed. The reason for the lack of increased caspase 3 activity in stimulated samples could potentially be its sensitivity to oxidation (40, 41), considering the high levels of oxidant produced by stimulated PMNs. Interestingly, we did not observe any significant effect of the classical inhibitor Z-VAD-fmk (100 µM) on the observed phenomenon of PS cell surface exposure on stimulated PMNs (data not shown). Similarly, the lack of effect of this inhibitor on the HOCl- and HOSCN-induced cell death of endothelial cells was reported by Lloyd et al. (40). Nevertheless, the PMN cell death observed in our study cannot be classified as conventional apoptosis. However, the very limited signal from phosphorylated RIP1 does not suggest a typical process of necroptosis either. What is interesting is the phenomenon of the rather significant changes in total Rip1 expression in stimulated PMNs independent of MPO; however, we did not find supporting literature that would clarify this phenomenon. Different authors have extensively studied the effects of PMA on PMNs in the context of the type of cell death and have reported the PMA-mediated activation of various mechanisms and markers related to different types of regulated cell death, including apoptosis, autophagy, necroptosis, and NETosis (7, 14, 19, 20, 25, 42). Similarly to our results, Aratani and colleagues observed PS externalization in PMA-activated PMNs that was not associated with caspase 3 activation (20, 43). Moreover, Fadeel et al. reported the absence of caspase activations in PMA-stimulated PMNs in contrast to significant caspase activation in PMNs treated with Fas ligand (25). Similarly, Takei et al. (42) did not observe DNA fragmentation in PMA-stimulated PMNs. In contrast, other studies have shown the presence of typical apoptotic markers such as DNA fragmentation and caspase activation in PMNs stimulated by PMA (44, 45).

To further clarify the relation between the MPO-dependent membrane alterations of activated PMNs, markers characteristic of the process of autophagy as well as the post-translational modifications of histones, both suggested to be connected with NETosis, were analyzed (16). Interestingly, in other studies, neither autophagy nor NETosis were found to be generally connected with PS externalization. In the presented model system, the activation of an autophagic process would suggest significantly elevated levels of LC3II fragments. However, the lack of p62 downregulation and, indeed, even the observed increase in p62 levels do not support the typical process of autophagy connected with the degradation of p62-containing aggregates of ubiquitylated proteins. In contrast, this could signify the accumulation of p62-containing aggregates of ubiquitylated proteins in activated dying PMNs. Interestingly, the increases in LC3-II and p62 levels were identical in WT and MPO^-/-^ PMNs and do not suggest a connection with PS surface exposure. In addition, autophagic processes were analyzed using a commercial kit employing the fluorescence substrate Cyto-ID, a cationic amphilic tracer dye labeling autophagic compartments, which did not reveal any positive results upon the stimulation of PMNs either from WT or MPO-deficient mice (data not shown). Finally, the citrullination of histone H3 independent of PMN activation was observed in our model in both WT and MPO^-/-^ PMNs. However, the surface presence of citrullinated histones increased significantly after both the PMA and OST activation of WT PMNs. Interestingly, this phenomenon was also observed in MPO^-/-^ PMNs, suggesting that the externalization of citrullinated histone H3 was not dependent on MPO. Histone citrullination in PMNs is connected with NETosis. MPO is suggested to be involved in NETosis that is dependent on NADPH oxidase-mediated ROS formation in PMNs (36, 37, 46–49). Metzler et al. showed that PMNs from a completely MPO-deficient donor did not form NETs (36). PMNs from partially MPO-deficient donors made NETs, while PMNs pretreated with the MPO inhibitor ABAH displayed a significant delay in NET formation (36). Interestingly, MPO-mediated protein translocation across the membrane is supposed to be dependent on MPO protein, but not on MPO activity (37).

This brings us to the question of the mechanism by which MPO contributes to the observed alteration of cell surface PS and membrane integrity. Regarding MPO enzymatic activity, no significant effect of 4-ABAH on the cell surface PS or membrane integrity was found. However, despite the application of a rather high concentration of 4-ABAH, we did not achieve the absolute inhibition of peroxidase activity in PMNs, as was shown by CL, and some minute level of MPO activity could have remained. Interestingly, externally added HOCl did not induce PS externalization. Interestingly again, a similar phenomenon was observed in HOCl-treated endothelial cells when HOCl concentrations of 25-100 µM did not induce PS surface exposure but direct permeabilization of the cell (40). Lower concentrations of HOCl (5 – 10 µM) significantly increased annexin V binding after 1h of incubation, though not after 2 h (41). This can be related to the very quick degradation of HOCl in complex cell culture media, when more than 70% of HOCl added in a concentration of 200µM is already lost after 15 min (40). Similarly to our study, Metzler et al. did not observe the induction of NETosis by the addition of extracellular HOCl (37). However, it should be noted that 4-ABAH did not completely block MPO activity, either in the study by Metzler or in ours.

The lack of an effect of the MPO inhibitor 4-ABAH on PS externalization allows us to speculate about a role for MPO which is independent of enzymatic activity in this process, one which could be connected with the binding of MPO to negatively charged membrane anionic phospholipids. The importance of these electrostatic and hydrophobic interactions was already suggested for cytochrome c, a highly cationic heme protein involved in cell death machinery (50). Importantly, we have, together with other authors, previously shown that MPO binds avidly to different negatively charged biomolecules, including glycosaminoglycans, components of the extracellular matrix, the surfaces of PMNs, erythrocytes, and platelets (39, 51–54). The binding of MPO to the PMN membrane could affect membrane stability and the translocation of PS independently of MPO enzymatic activity. Metzler et al. suggested that MPO is required independently of enzymatic activity for the release of neutrophil elastase from azurophilic granules into the cytosol, leading to the localization of neutrophil elastase in the nucleus after PMA stimulation (37). Conversely, our data showed the increased citrullination of histone H3 and the increased surface expression of these citrullinated histones in both WT and MPO^-/-^ PMNs.

In conclusion, these results suggest that MPO plays an important role in regulating the course of PMN-regulated cell death. Nevertheless, further work evaluating the time course of the appearance of apoptotic PMNs is required to confirm the role of MPO in regulated cell death and to determine whether such defective functions of PMNs are involved in the pathology of various inflammatory conditions. One limitation of this study is the natural difference among the contents of MPO in mouse and human PMNs, since human PMNs have a significantly higher content of MPO (19, 29) and thus could affect the fate of PMNs more profoundly. Another limitation of this study is the use of PMNs from constitutive MPO KO mice, which may have altered expressions of various proteins to compensate for the deficient MPO, which may, in turn, affect the overall outcome. Knowledge gained from this research will help to determine more extensively the biological functions of MPO in inflammatory lung disease and will aid in the development of potential pharmacological treatments for both acute and chronic inflammation-mediated diseases.

## Ethical Statement

The animal study was reviewed and approved by Animal Care and Use Committee, The Czech Academy of Sciences of the Czech Republic.

## Data Availability Statement

The raw data supporting the conclusions of this article will be made available by the authors, without undue reservation.

## Author Contributions

SK, AKo, MC, and LK performed the experiments and analyzed the data. SK, AKo, MC, AKl, and LK contributed to the conception of the study, to the experimental design, and to the writing of the manuscript. AKl and LK contributed to funding acquisition and to the review and editing of the manuscript. All authors contributed to the article and approved the submitted version.

## Funding

This work was supported by institutional support from the Institute of Biophysics of the Czech Academy of Sciences and by the European Regional Development Fund - Project INBIO (No. CZ.02.1.01/0.0/0.0/16_026/0008451).

## Conflict of Interest

The authors declare that the research was conducted in the absence of any commercial or financial relationships that could be construed as a potential conflict of interest.

## Publisher’s Note

All claims expressed in this article are solely those of the authors and do not necessarily represent those of their affiliated organizations, or those of the publisher, the editors and the reviewers. Any product that may be evaluated in this article, or claim that may be made by its manufacturer, is not guaranteed or endorsed by the publisher.

## Supplementary Material

Supplementary Figure 1 Representative scatterplots from flow-cytometric analysis illustrating the data shown in **Figure 1** presenting Annexin V^+^ and cell death (PI^+^) of PMNs from WT and MPO deficient mice after the induction of oxidative burst by PMA, OST, and fMLP for 90 min **(A–C)** and 180 min **(D–F)**. PMNs (3x 10^6^ cells/ml) isolated from BALF of WT and MPO^-/-^ mice were stimulated with PMA (80 nM), OST (1:10 MOI) and fMLP (1.14 μM) for 90 and 180 min. Cells were classified as either viable cells (lower left quartile, Annexin V^-^/PI^-^), cells with a detectable expression of PS (lower right quartile, Annexin V^+^/PI^-^), or dead cells with permeable membranes (upper left and right quartile, PI^+^).

Supplementary Figure 2 Absence of a significant effect of 4-ABAH on Annexin V^+^ and cell death (PI^+^) of PMNs from WT mice after induction of oxidative burst by PMA for 90 min **(A–C)** and 180 min **(D–F)**. PMNs (3x 10^6^ cells/ml) isolated from BALF of WT mice were pre-treated for 10 min with 4-ABAH (500 µM) followed by stimulation with PMA (80 nM) for 90 and 180 min. Next, cells were evaluated by flow cytometry and numbers of viable (ANX^-^/PI^-^), Annexin V^+^ (ANX^+^/PI^-^), and dead cells (PI^+^, both ANX^+^/PI^+^ and ANX^-^/PI^+^) were determined. n = 3-4 mice per group. Data are presented as median, 25, and 75 percentiles (the box) and 5 and 95 percentiles (the whiskers), and values below and above the whiskers are drawn as individual dots. * shows statistically significant difference (p < 0.05) compared with untreated control (Ctrl).

Supplementary Figure 3 Effects of 4-ABAH on spontaneous and PMA-, OST-, and fMLP-activated ROS production by PMNs isolated from WT and MPO^-/-^ mice **(A)** and the concentration-dependent effects of 4-ABAH on oxidative burst (ROS production) induced by PMA, OST, and fMLP in PMNs isolated from WT **(B)** and MPO^-/-^ **(C)** mice. **(A)** Cells (1.25 x 10^6^ cells/ml) isolated from BALF of WT and MPO^-/-^ mice were stimulated with PMA (800 nM), OST (1:10 MOI) and fMLP (1.14 μM) in the presence of luminol, and CL measurements were performed for 120 min at 37°C. Spontaneous CL measurements in samples containing all other substances, but none of the activators, were also determined. **(A, B)** Cells (1.25 x 10^6^ cells/ml) isolated from BALF of WT and MPO^-/-^ mice were pre-treated with 4-ABAH inhibitor (250 and 500 µM) for 10 min, followed by stimulation with PMA (800 nM), OST (1:10 MOI) and fMLP (1.14 μM) in the presence of luminol. CL measurement was performed for 120 min at 37°C. Spontaneous CL measurements in samples containing all other substances, but none of the activators, were also performed. Values of the integral measurement of the CL signal (area under the curve), which represents the total ROS production by PMNs, were determined. Values represent mean ± SEM (A - n = 5-6, B and C - n = 4). *Shows statistically significant difference (p < 0.05) compared to untreated control (Ctrl). # shows statistically significant difference (p < 0.05) between MPO^-/-^ (MPO-KO) and wild-type (WT).

## Abbreviations

4-ABAH, 4-Aminobenzoic acid hydrazide; Ac-DEVD-AMC, N-Acetyl-Asp-Glu-Val-Asp-7-amido-4-Methylcoumarin; ANX, Annexin V; BALF, bronchoalveolar lavage fluid; CL – chemiluminescence; Ctrl – control; fMLP, N-Formylmethionyl-leucyl-phenylalanine; GAPDH, Glyceraldehyde 3-phosphate dehydrogenase; HBSS, Hanks’ Balanced Salt solution; HEPES, 4-(2-hydroxyethyl)-1-piperazineethanesulfonic acid; LC3- the microtubule-associated proteins 1A/1B light chain 3B;LPS – lipopolysaccharide; MPO, Myeloperoxidase; MPO-KO, MPO-deficient mice; NADPH oxidase, nicotinamide adenine dinucleotide phosphate oxidase; NETs, neutrophil extracellular traps; OD, optical density; OST, opsonized streptococcus mutans; PMA, 12-myristate 13-acetate; PBS, phosphate-buffered saline; PI, propidium iodide; PMNs, Polymorphonuclear neutrophils; PS – phosphatidylserine; RIP1, Receptor-interacting serine/threonine-protein kinase 1; ROS, reactive oxygen species; RT, room temperature; SDS, sodium dodecyl sulfate; TNF-α, tumor necrosis factor α; UV – ultraviolet; WT, wild-type.
