# Supplementary figures and images for: Myeloperoxidase Deficiency Alters the Process of the Regulated Cell Death of Polymorphonuclear Neutrophils
Source: Front Immunol. 2022 Feb 8;13:707085. doi: 10.3389/fimmu.2022.707085 (PMC8860816; doi:10.3389/fimmu.2022.707085)

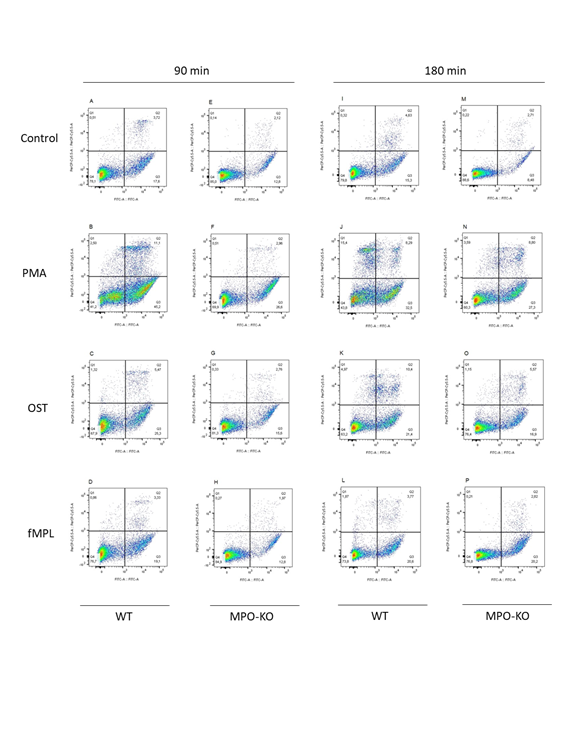

Supplement: Supplementary Figure 1 — Representative scatterplots from flow-cytometric analysis illustrating the data shown in Figure 1 presenting Annexin V+ and cell death (PI+) of PMNs from WT and MPO deficient mice after the induction of oxidative burst by PMA, OST, and fMLP for 90 min (A–C) and 180 min (D–F). PMNs (3x 10^6^ cells/ml) isolated from BALF of WT and MPO-/- mice were stimulated with PMA (80 nM), OST (1:10 MOI) and fMLP (1.14 μM) for 90 and 180 min. Cells were classified as either viable cells (lower left quartile, Annexin V-/PI-), cells with a detectable expression of PS (lower right quartile, Annexin V+/PI-), or dead cells with permeable membranes (upper left and right quartile, PI+). [file Image_1.tif]

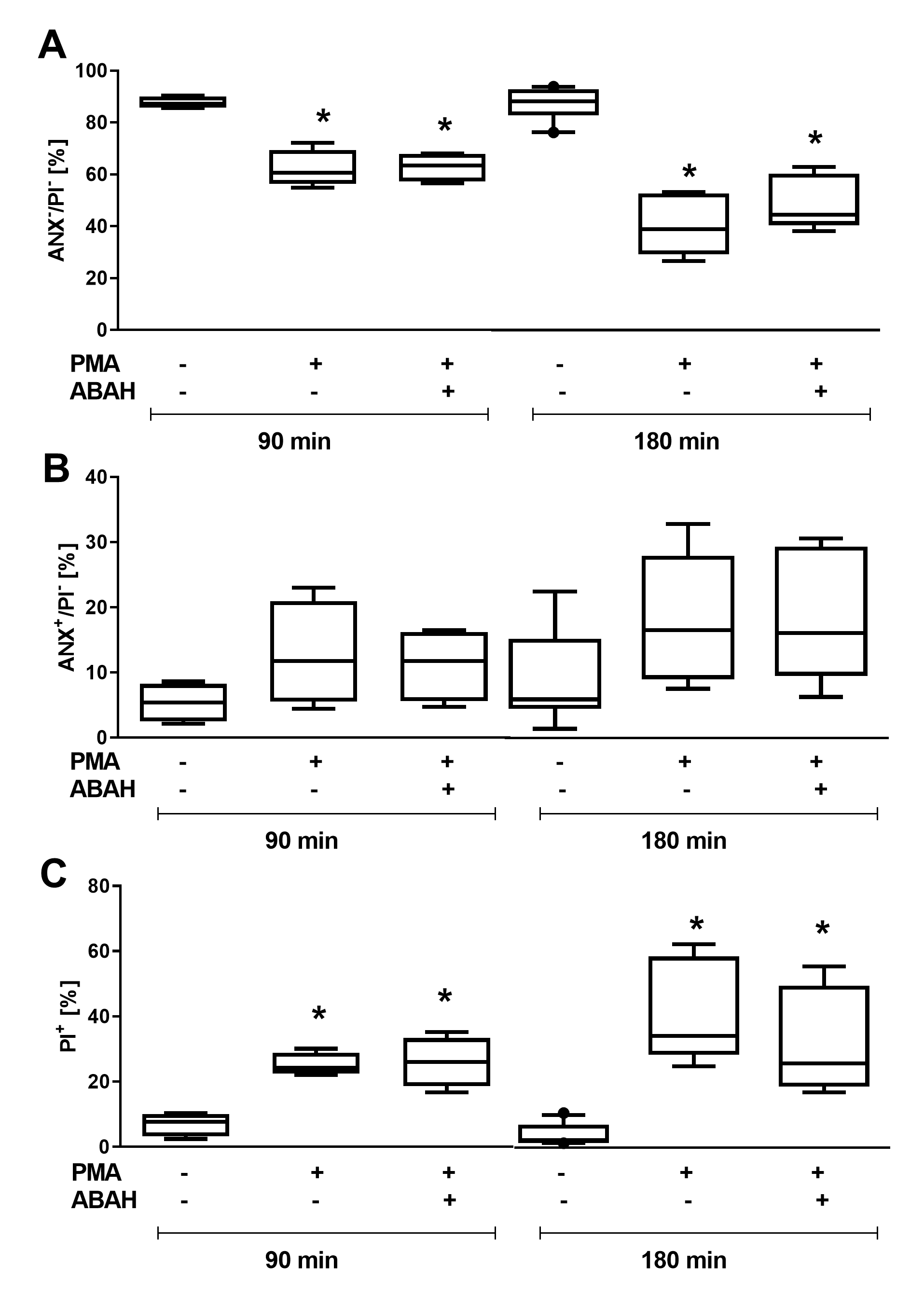

Supplement: Supplementary Figure 2 — Absence of a significant effect of 4-ABAH on Annexin V+ and cell death (PI+) of PMNs from WT mice after induction of oxidative burst by PMA for 90 min (A–C) and 180 min (D–F). PMNs (3x 10^6^ cells/ml) isolated from BALF of WT mice were pre-treated for 10 min with 4-ABAH (500 µM) followed by stimulation with PMA (80 nM) for 90 and 180 min. Next, cells were evaluated by flow cytometry and numbers of viable (ANX-/PI-), Annexin V+ (ANX+/PI-), and dead cells (PI+, both ANX+/PI+ and ANX-/PI+) were determined. n = 3-4 mice per group. Data are presented as median, 25, and 75 percentiles (the box) and 5 and 95 percentiles (the whiskers), and values below and above the whiskers are drawn as individual dots. * shows statistically significant difference (p < 0.05) compared with untreated control (Ctrl). [file Image_2.tif]

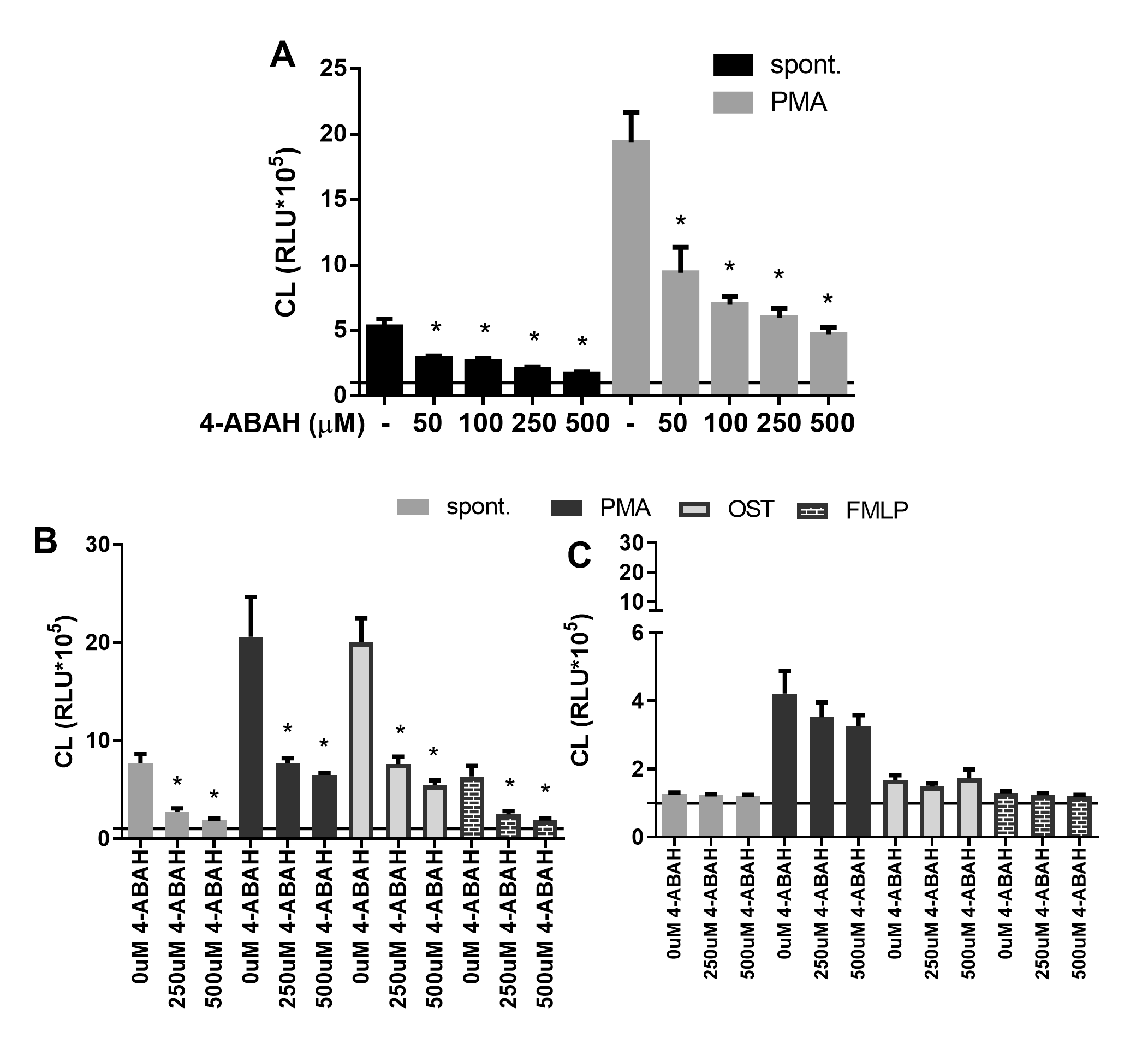

Supplement: Supplementary Figure 3 — Effects of 4-ABAH on spontaneous and PMA-, OST-, and fMLP-activated ROS production by PMNs isolated from WT and MPO-/- mice (A) and the concentration-dependent effects of 4-ABAH on oxidative burst (ROS production) induced by PMA, OST, and fMLP in PMNs isolated from WT (B) and MPO-/- (C) mice. (A) Cells (1.25 x 10^6^ cells/ml) isolated from BALF of WT and MPO-/- mice were stimulated with PMA (800 nM), OST (1:10 MOI) and fMLP (1.14 μM) in the presence of luminol, and CL measurements were performed for 120 min at 37°C. Spontaneous CL measurements in samples containing all other substances, but none of the activators, were also determined. (A, B) Cells (1.25 x 10^6^ cells/ml) isolated from BALF of WT and MPO-/- mice were pre-treated with 4-ABAH inhibitor (250 and 500 µM) for 10 min, followed by stimulation with PMA (800 nM), OST (1:10 MOI) and fMLP (1.14 μM) in the presence of luminol. CL measurement was performed for 120 min at 37°C. Spontaneous CL measurements in samples containing all other substances, but none of the activators, were also performed. Values of the integral measurement of the CL signal (area under the curve), which represents the total ROS production by PMNs, were determined. Values represent mean ± SEM (A - n = 5-6, B and C - n = 4). *Shows statistically significant difference (p < 0.05) compared to untreated control (Ctrl). # shows statistically significant difference (p < 0.05) between MPO-/- (MPO-KO) and wild-type (WT). [file Image_3.tif]
